# While reinforcing cell cycle arrest, rapamycin and Torins suppress senescence in UVA-irradiated fibroblasts

**DOI:** 10.18632/oncotarget.17827

**Published:** 2017-05-11

**Authors:** Olga V. Leontieva, Mikhail V. Blagosklonny

**Affiliations:** ^1^ Cell Stress Biology, Roswell Park Cancer Institute, Buffalo, NY, USA

**Keywords:** mTORC1, rapalogs, sirolimus, aging, cancer, UVA

## Abstract

Sunlight predisposes to skin cancer and melanomas. Ultraviolet A (UVA), a long wave component of sunlight, can reach dermal fibroblasts. Here we studied UVA-induced senescence in human fibroblasts *in vitro*. It is known that senescence occurs, when cell cycle is arrested, but mTOR is still active, thus converting arrest to senescence (geroconversion). We showed that, while arresting cell cycle, UVA did not inhibit mTOR, enabling geroconversion. In UVA-treated cells, mTOR remained fully active. Rapamycin and Torins 1/ 2 prevented UVA-induced senescent phenotype, although they further re-enforced cell cycle arrest. Given that senescent stromal fibroblasts support tumorigenesis, we envision that mTOR inhibitors may potentially be used to prevent sunlight-caused tumors as well as skin photo-aging.

## INTRODUCTION

Sunlight that reaches our skin consists of infrared, visible, ultraviolet A (UVA) and ultraviolet B (UVB) light. Ultraviolet B targets epidermis and damages DNA in keratinocytes and melanocytes, thus initiating carcinogenesis [1-6].

Long wavelength ultraviolet light UVA, which is less energetic than UVB, is a main component of UV radiation we exposed to. UVA deeply penetrates into the skin (the dermal layer), targeting dermal fibroblasts. *In vitro*, UVA can induce senescence in fibroblasts [7-10]. Senescent fibroblasts can promote carcinogenesis and also contribute to photo-aging [11].

In cell culture, induction of senescence includes two steps: cell cycle arrest followed by geroconversion [12, 13]. During geroconversion cells become hypertrophic (large cell morphology) and hyper-functional (hyper-secretory phenotype or SASP), acquire β- galactosidase (β-Gal) staining, a marker of lysosomal hyperfunction, and lose re-proliferative potential [12-17]. Geroconversion is in part mTORC1-dependent and is partially suppressed by rapamycin and other rapalogs [18-34]. Pan-mTOR inhibitors, which inhibit both rapamycin-sensitive and -insensitive activities of mTORC1 [35,36], further suppress geroconversion [31-33, 37].

Here we showed that UVA caused senescence in normal human WI38t fibroblasts and primary adult dermal murine fibroblasts. Rapamycin and pan-mTOR inhibitors prevented UVA-induced senescent morphology. mTOR inhibitors did not abrogate cell cycle arrest and in contrast reinforced it. Thus, mTOR inhibitors suppressed geroconversion of UVA-arrested fibroblasts.

## RESULTS

### Torins and rapamycin potentiate cell cycle arrest caused by UVA

**Figure 1:**
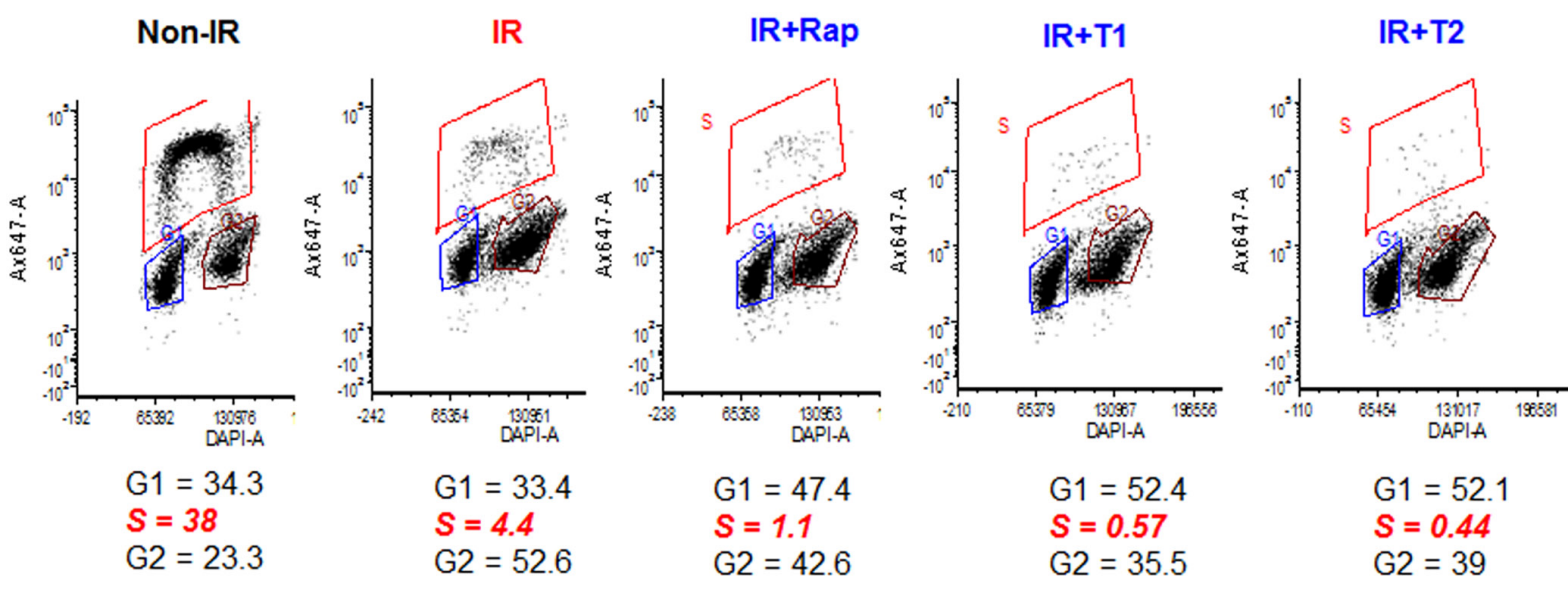
mTOR inhibitors re-enforce arrest caused by UVA in WI38t fibroblasts Fibroblasts were irradiated with 8 J/cm^2^ of UVA (IR) and were left untreated or treated with either rapamycin (Rap, 5 nM), Torin 1 (T1, 30 nM) or Torin 2 (T2, 30 nM) immediately after irradiation. Non-irradiated (non-IR) cells serve as proliferation control. 24 h after irradiation cells were pulsed with 10 µM Edu for 1 h, trypsinized, fixed in 4% paraformaldehyde, stained for Edu and DAPI as described in Methods and analyzed by flow cytometry. Note: percentage of EDU-positive (S phase) cells is measured to estimate S phase. G1 and G2 were estimated by DAPI staining, so % of G1+G2+S is not necessarily 100.

In pilot experiments, we selected 8-10 J/cm2 of UVA irradiation to induce senescence in human WI38t fibroblasts. As shown in Figure 1, irradiation caused predominately G2 arrest, decreasing the number of cells in S-phase. We next investigated effects of low concentrations of mTOR inhibitors on UVA-induced arrest: 5 nM rapamycin and 30 nM Torin 1 and Torin 2, an optimal non-toxic gerosuppressive concentration of Torins [37]. mTOR inhibitors further decreased S phase fraction in UVA-arrested cells (Figure 1). mTOR inhibitors shifted arrest from G2 to G1, so cells were arrested equally in G1 and G2 with rapamycin and predominantly in G1 with Torins.

### Torins and rapamycin prevent UVA-induced senescence in WI38t fibroblasts

**Figure 2:**
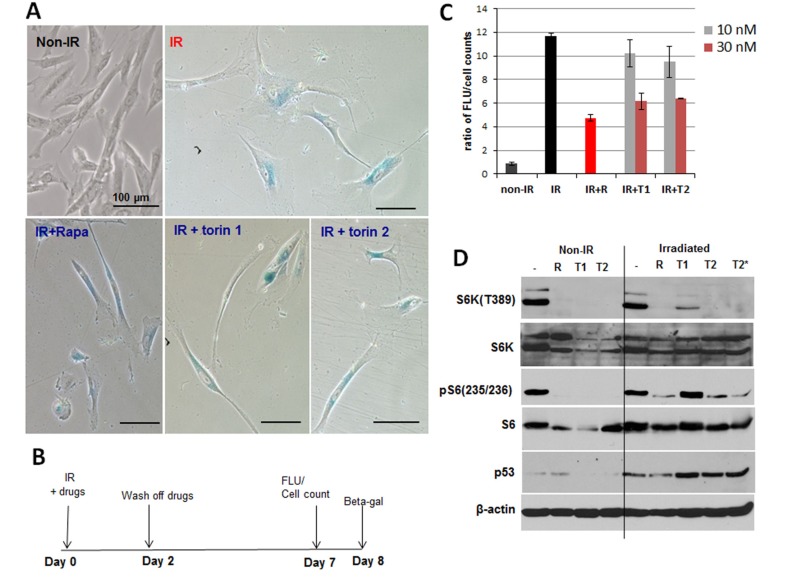
mTOR inhibitors suppress senescent phenotype induced by UVA in WI38t fibroblasts **A.** SA-β-gal staining. WI38t cells were irradiated with 8 J/cm^2^ (IR) and treated with mTOR inhibitors as shown in schema **B.** 8 days after irradiation cells were stained for SA-β-gal and microphotographed. Non-IR – non-irradiated cells serve as control. **C.** mTOR inhibitors reduce metabolic activity of senescent WI38t fibroblasts. Cells were induced to senesce by UVA irradiation and treated as shown in schema (B). 7 days after irradiation cellular metabolic activity was measured using CellTiter Blue reagent as described in Methods, followed by cell counts. Data present a ratio of fluorescence units (FLU) to cell counts, i.e. fluorescence units per cell. Bars are mean ± SD. R – rapamycin 5 nM; T1 – Torin 1; T2 – Torin 2. **D.** Immunoblot analysis. WI38t cells were irradiated or not (non-IR) with 8 J/cm^2^ and drugs were added immediately after irradiation. 24 h later cells were lysed and immunoblotting was performed with indicated antibodies.

**Figure 3:**
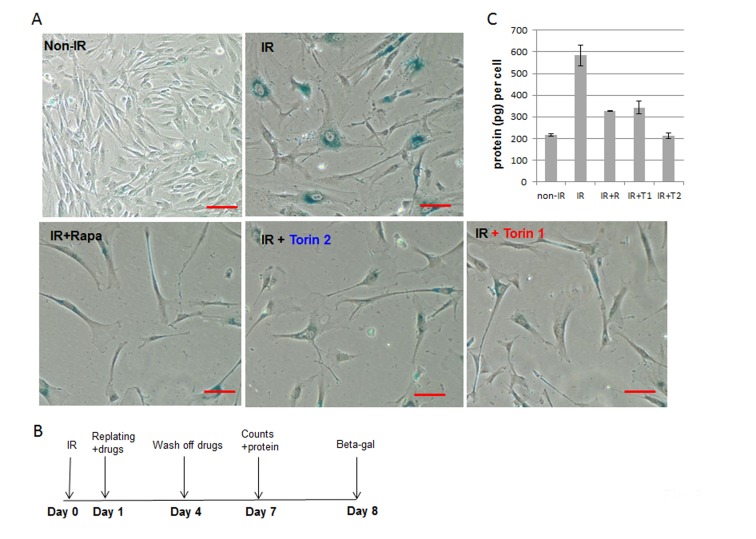
mTOR inhibitors suppress senescent morphology and hypertrophy in WI38t fibroblasts, when treatment was delayed by 24 h after irradiation **A.** SA-β-gal staining. Cells were irradiated with 10 J/cm^2^ (IR) and treated as shown in schema **B.** 8 days after irradiation cells were stained for SA-β-gal and microphotographed; bar- 100 µm; **C.** Cells were treated as in (B). 7 days after irradiation, cells were counted and lysed followed by measuring protein amounts. Data present protein per cell. Non-IR – non-irradiated control cells.

Irradiated WI38t cells developed senescent phenotype: enlarged cell morphology and SA-β-gal staining (Figure 2A). Rapamycin and Torins 1 and 2 prevented hypertrophic morphology , when drugs were added immediately after irradiation (Figure 2) or when cells were additionally pre-treated for 1 day before irradiation and treated after irradiation too (Supplementary Figure S1). UVA-irradiated fibroblasts treated with mTOR inhibitors remained smaller/thinner in size compared to irradiated cells (Figure 2A and Supplementary Figure S1). For all further studies, we chose to treat cells after irradiation (as depicted in schemas in Figure 2B and 3B).

Since one of the features of senescent cells is hyperfunction, we compared metabolic rate of irradiated senescent WI38t fibroblasts and control non-irradiated cells by measuring reduction of resazurin to fluorescent resorufin. Resazurin was added to cultures 6-7 days after irradiation and fluorescence of resorufin was measured (Figure 2B, 2C and Supplementary Figure S2). Then, cells were trypsinized and counted in hemocytometer and ratios of fluorescence units to cell numbers were calculated. In non-irradiated cultures, rapamycin and Torins did not affect ratios of fluorescence intensities to cell numbers (Supplementary Figure S2), indicating that metabolic rate per cell was constant. In irradiated cells, fluorescence intensity per cell was high (Figure 2C) and 5nM rapamycin and 30 nM Torin 1 and 2 decreased metabolic activity of irradiated cells (Figure 2C).

### While decreasing mTOR activity, mTOR inhibitors did not decrease p53 in UVA-irradiated cells

UVA induced p53 (Figure 2D), which is known to mediate cell cycle arrest caused by irradiation. Rapamycin did not abrogate p53 induction and Torins even potentiated it. So suppression of senescent phenotype by mTOR inhibitors is not due to abrogation of the pathway leading to cell cycle arrest. The arrest pathway remained intact (Figure 2D), consistent with cell cycle distribution (Figure 1). We next investigated the geroconversion pathway. UVA did not inhibit phospho-S6K (T389) and phospho-S6(S235/236) (Figure 2D). Treatment with rapamycin and 30 nM Torin 2 resulted in sustained inhibition of phospho-S6K and S6 (S235/236). In agreement with previous results [37], Torin 1 was less potent than Torin 2 (Figure 2D). Curiously, mTOR inhibitors including rapamycin were less effective in inhibiting S6 phosphorylation in UVA-irradiated cells compared to non-irradiated control cells (Figure 2D).

Rapamycin and Torins suppressed geroconversion of UVA-irradiated WI38t fibroblasts even when treatment began 24 h after radiation (Figure 3B). Treated cells remained morphologically smaller (Figure 3A) and less hypertrophic as indicated by reduced amount of protein per cell (Figure 3C).

### Torins and rapamycin prevent UVA-induced senescence in primary adult mouse skin fibroblasts

**Figure 4:**
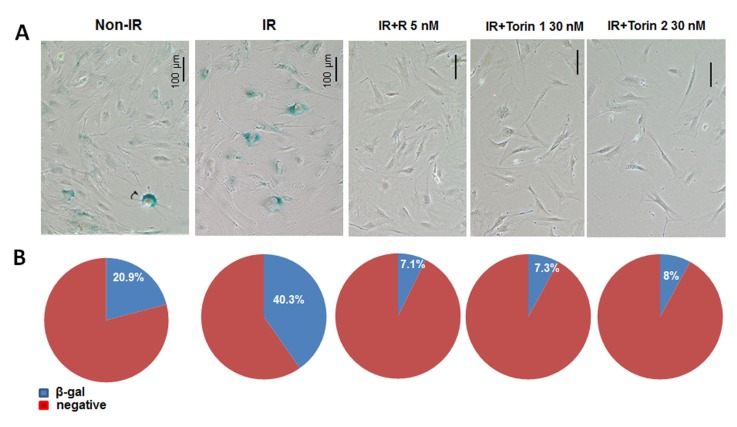
mTOR inhibitors suppress senescent morphology in UVA-irradiated primary adult mouse skin fibroblasts **A.** Cells were pre-treated with mTOR inhibitors for 3h and then irradiated with 10 J/cm^2^ (IR). Drugs were re-added after irradiation. Four days after irradiation cells were stained for SA-β-gal. Non-IR – non-irradiated control; IR – irradiated, R – rapamycin ; T1 – Torin 1; T2 – Torin 2 . **B.** Numbers of β-gal positive and negative cells were counted in 4-6 fields for each sample. Counts were combined and percentage of β-gal positive cells was calculated.

**Figure 5:**
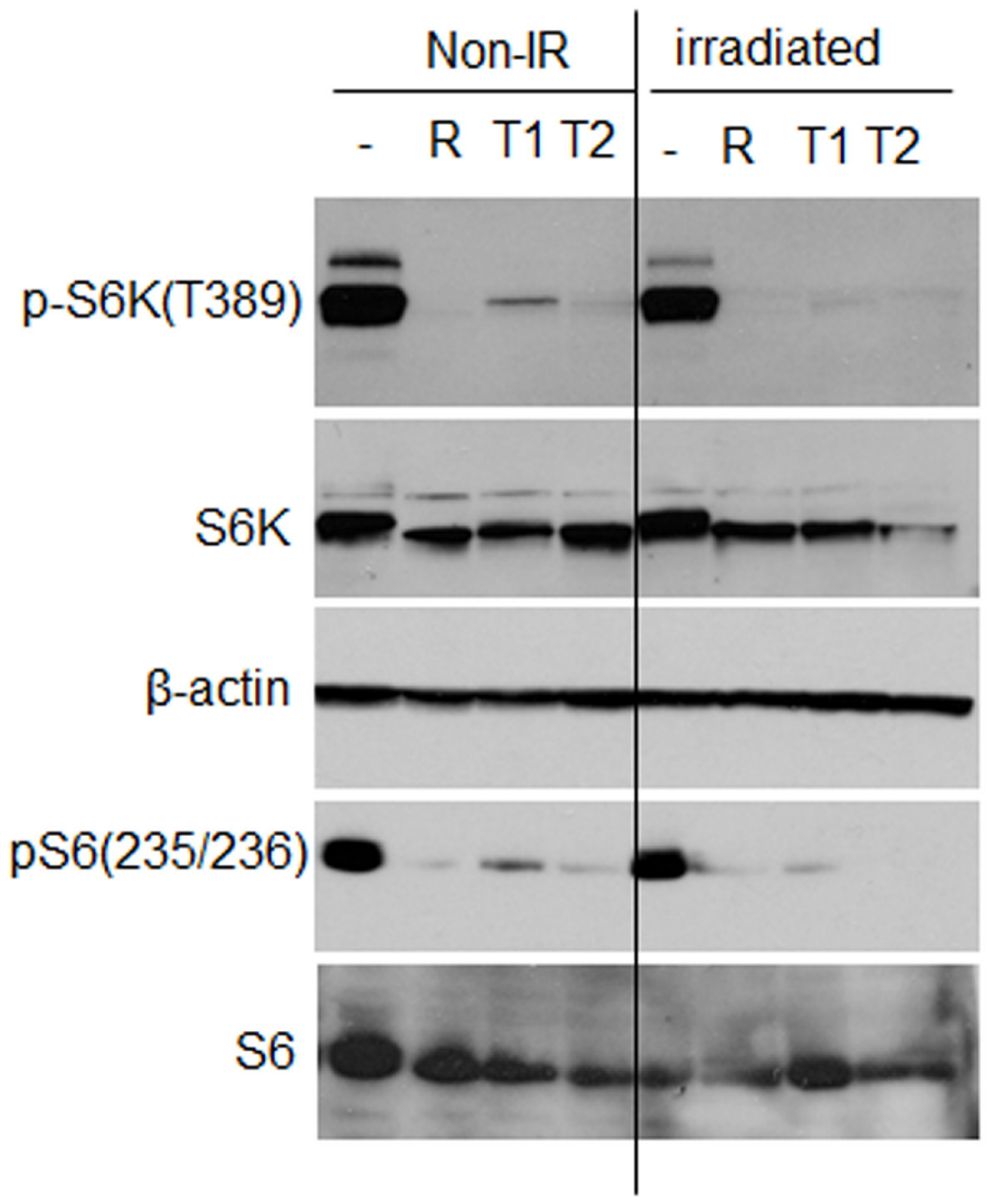
UVA irradiation does not inhibit mTOR pathway in primary adult mouse fibroblasts Immunoblot analysis. Primary adult murine fibroblasts were pre-treated with mTOR inhibitors for 3 h and then irradiated with 10 J/cm^2^. Drugs were re-added and cells were lysed 24 h after irradiation. Non-IR – non-irradiated control; R – rapamycin (5 nM); T1 - Torin 1 (30 nM); T2 – Torin 2 (30 nM).

We extended our study to primary adult mouse skin fibroblasts. Primary skin fibroblast cultures were isolated from murine skin and used at early passage (p.2) since these cells undergo replicative senescence when cultured *in vitro* by passage 5. Primary skin fibroblasts were pre-treated with mTOR inhibitors for 3 hours, then irradiated with UVA and treatment continued for 4 days followed by SA-β-gal staining (Figure 4A). SA-β-gal-positive cells were counted in 4-6 fields of each condition and percent of senescent cells was calculated (Figure 4B). Non-irradiated control cell culture had a noticeable number of senescent cells (∼ 21%), which is not unusual for primary cultures. Four days after irradiation, number of senescent cells doubled in control irradiated cultures. mTOR inhibitors decreased fraction of SA-β-gal positive enlarged cells in irradiated cultures. As in the case of WI38t cells, these effects were associated with inhibition of mTORC1 pathway as evidenced by inhibited phosphorylation of S6K at T389 and S6 at S235/236 (Figure 5).

## DISCUSSION

Here we showed that UVA caused cell cycle arrest followed by mTOR-dependent geroconversion, which could be suppressed by rapamycin and Torin 1 and 2. mTOR inhibitors prevented only the second step of senescence program: geroconversion. Cell cycle arrest caused by UVA was not abrogated. Furthermore, the arrest was re-enforced. mTOR inhibitors by themselves slow down cell cycle progression. It is important to emphasize because of the common misunderstanding of the difference between cell cycle arrest and senescence [12, 13]. mTOR inhibitors arrest cell cycle, yet inhibit geroconversion in arrested (quiescent) cells. Cells remain quiescent, not senescent. Quiescent cells retain the ability to re-proliferate. So mTOR inhibitors inhibit proliferation but may preserve re-proliferative potential, which can be evident when cells are re-stimulated to proliferate [12,13, 31]. We emphasize again that mTOR inhibitors do not abrogate senescent arrest, do not re-activate cell cycle, do not stimulate proliferation. They preserve the potential to re-proliferate, when cell cycle is re-activated by removing CDK inhibition [12, 13, 31].

Suppression of geroconversion in UVA-treated fibroblasts has several implications. First, by inducing senescence in dermal fibroblasts, UVA may create pro-carcinogenic micro-environment to promote premalignant keratinocytes and melanocytes. In fact, hyper-functional senescent cells secrete tumor-promoting molecules and support carcinogenesis [38-43]. By suppressing development of UV-induced senescent phenotype in stromal fibroblasts, mTOR inhibitors may prevent UV-induced tumors. In fact, rapamycin suppress UVB-induced skin cancer in mice [44], decrease clusters of premalignant cells with mutant p53 after UVA+UVB-radiation [45]. Although not much is known about the effect of mTOR inhibitors on UV-induced carcinogenesis, it is recognized that rapamycin prevents cancer by other carcinogens [46] and spontaneous cancer in animals and humans [47-61]. Also, rapamycin prevents TPA-induced skin tumors [62]. Noteworthy, TPA can activate mTOR and induce cellular senescence in certain cell types [63]. Rapamycin prevents cancer in a wide variety of cancer-prone murine models [64-70]. Rapamycin and everolimus prevent skin cancer in humans: namely, in transplant patients receiving rapamycin (sirolimus) and everolimus [57-61]. mTOR inhibitors are very attractive chemopreventive modality, given their systemic anti-aging effects [54, 55].

Finally, by reducing cellular senescence, rapamycin may be considered to prevent photo aging. Rapalogs (rapamycin and everolimus) can be used not only systemically but also topically. Rapalog-based creams are expected not to interfere with sun tanning and vitamin D3 synthesis.

## MATERIALS AND METHODS

### Cell lines and reagents

WI38-tert (WI38t) fibroblasts were provided by Dr. Eugene Kendal (Roswell Park Cancer Institute, Buffalo, NY) and described previously [71]. WI38t cells were cultured in DMEM, supplemented with 10% FBS and pen/strep. Primary adult mouse skin fibroblasts were a kind gift from Dr. G. Paragh laboratory (Roswell Park Cancer Institute, Buffalo, NY). Primary fibroblasts were maintained in DMEM supplemented with 10% FBS, pen/strep and antibiotic-antimycotic (ANTI-ANTI; Thermo Fisher Scientific, Grand Island, NY).

Rapamycin was purchased from LC laboratories (Woburn, MA). Torin 1 and Torin 2 were from Selleckchem (Houston, TX). Stock solutions were prepared in DMSO.

### Senescence induction

Cells were induced to senesce by exposure to UVA1 radiation, which makes up to 75% of UVA rays. UVA1 irradiation was produced by a UVP transilluminator with 5x8W Hitachi F8T5 UVA1 fluorescent light tubes. The spectral output was determined by a STS-UV-L-25-400-SMA STS Microspectrometer (Ocean Optics Inc). UVA1 dose was determined using an International light radiometer/photometer (IL1400A).

Immediately before irradiation complete medium was replaced with DMEM without phenol red/5%FBS. Proliferating cells were irradiated for an interval needed to deliver 8 or 10 J/cm^2^ of UVA1 rays; control non-irradiated (mock irradiated) cells were placed in the same hood for an identical interval but not being irradiated. Then cells were treated with mTOR inhibitors or left untreated and incubation was continued in complete growth medium.

Irradiated cells were treated with mTOR inhibitors as described in Figure legends.

After several days, cells were scored for senescence markers, such as SA-β-gal activity, hypertrophy (displayed as protein amount per cell) and increased metabolic activity (rate of reduction of resazurin to resorufin).

### Cell cycle distribution and DNA synthesis

To determine rate of DNA synthesis, cells were pulsed with 10 µM EdU (5-ethynyl-2’-deoxyuridine; Thermo Fisher Scientific, Grand Island, NY) for 1 hour. Labeled cells were trypsinized, washed in PBS and fixed in 4% paraformaldehyde for 20 min at RT and washed in PBS. Fixed cells were further stained by incubating in the staining cocktail (4 mM CuSO4, 100 mM sodium ascorbate and 4.8 µM Alexa Flour 647 Azide triethylammonium (Thermo Fisher Scientific)) for 20 min at RT followed by washes in PBS. Cells were re-suspended in PBS and counterstained with 5 µg/ml of DAPI. Analyses were performed on flow cytometer Fortessa B at Roswell Park Cancer Institute FACS facility, using BD FACS Diva Software (BD Biosciences). Data were collected for 20000 cells and analyzed with FCS Express 4 (De Novo Software, Glendale, CA).

SA-β-gal staining was performed using Senescence-galactosidase staining kit (Cell Signaling Technology, Danver, MA) according to manufacturer’s protocol. Photos were taken under bright field microscope.

### Immunoblot analysis

Whole cell lysates were prepared using boiling lysis buffer (1% SDS, 10 mM Tris.HCl, pH 74.). Protein concentrations were determined using BCA reagent (Thermo Scientific, Rockford, IL) and equal amounts of proteins were separated onto Criterion or mini gradient polyacrylamide gels (Bio-Rad, Hercules, CA) and transferred to PVDF membranes (Bio-Rad). The following primary antibodies were used: rabbit antibodies for phospho-S6 (Ser235/236), phospho-S6K(T389 ) and total S6K - were from Cell Signaling Biotechnology. Mouse anti-S6 antibody was from Cell Signaling Biotechnology. Anti-β-actin-HRP antibody was from Sigma-Aldrich (St. Louis, MO); mouse antibody for p53 (Ab-6) was from Oncogene Research products (La Jolla, CA). Secondary anti-rabbit and anti-mouse HRP-conjugated antibodies were from Cell Signaling Biotechnology as previously described [20] , [21] .

### Metabolic activity

Test was performed using CellTiter Blue reagent (Promega, Madison, WI). The resazurin dye (CellTiter Blue) is reduced to resorufin by metabolically active cells, resulting in the generation of a fluorescent product. 200 µl of CellTiter Blue was added per 1 ml of medium to the cells followed by incubation at 37°C, 5% CO2 for 3 h. Fluorescence was recorded at 560/590 nm. Then cells were counted and ratios of fluorescence units to cell numbers were calculated.

## SUPPLEMENTARY MATERIALS FIGURES


