# Supplementary material for: While reinforcing cell cycle arrest, rapamycin and Torins suppress senescence in UVA-irradiated fibroblasts
Source: Oncotarget. 2017 May 11;8(65):109848–56. doi: 10.18632/oncotarget.17827 (PMC5752566; doi:10.18632/oncotarget.17827)
Supplement: Supplementary file 1 [file oncotarget-08-109848-s001.pdf]

While reinforcing cell cycle arrest, rapamycin and Torins suppress senescence in UVA-irradiated fibroblasts

Supplementary Material

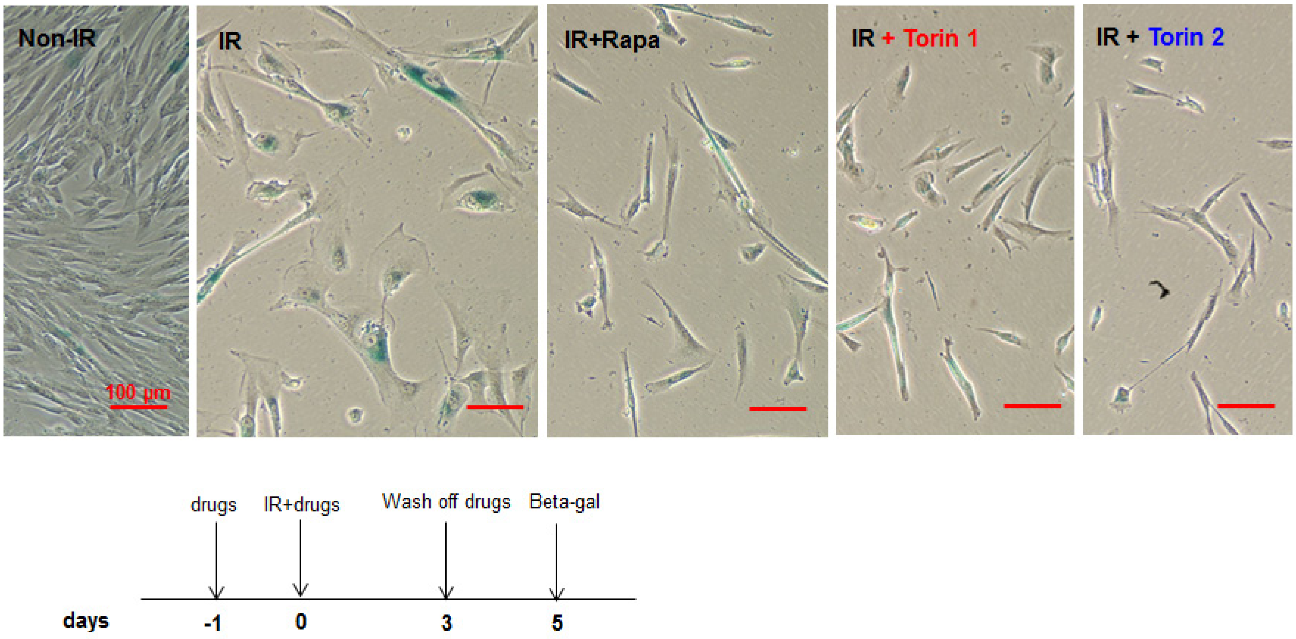

Supplementary Figure S1: SA-β-gal staining. Pre-treatment and posttreatment with rapamycin or torins prevented senescent morphology In WI38t fibroblasts, caused by irradiation with 8 J/cm2 of UVA1. Non-IR – non-irradiated cells; IR – irradiated; Rapa – rapamycin

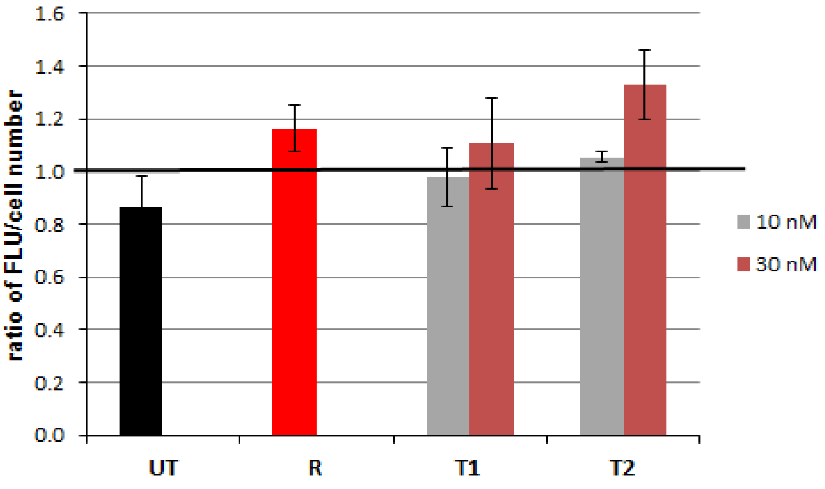

Supplementary Figure S2: Metabolic activity of WI38t fibroblasts treated with mTOR inhibitors. WI38t cells were treated with mTOR inhibitors for two days, then drugs were washed out. Metabolic rate was estimated using CellTiter Blue reagent as described in Methods. UT – untreated; R – rapamycin at 5 nM; T1 -torin 1; T2 – torin 2.
